# Characterization of the complete mitochondrial genome of *Quasilineus sinicus* Gibson, 1990 (Nemertea: Heteronemertea) and its phylogenetic implications

**DOI:** 10.1080/23802359.2022.2126287

**Published:** 2022-09-27

**Authors:** Chun-Yang Shen, Wei Xue, Chong Pang, Asem Alireza, Xiaonan Mao, Jiahui Han, Haonan Chen, Chunzheng Fu

**Affiliations:** a Department of Biology, Chengde Medical University, Chengde, Hebei Province, China; b Department of Chemical Engineering, Hebei Petroleum University of Technology, Chengde, Hebei Province, China; c Department of Pharmacology, Chengde Medical University, Chengde, Hebei Province, China; d Hainan Key Laboratory for Conservation and Utilization of Tropical Marine Fishery Resources, Hainan Tropical Ocean University, Sanya, Hainan Province, China; e Institute of Sericulture, Chengde Medical University, Chengde, Hebei Province, China

**Keywords:** *Quasilineus sinicus*, Nemertea, mitogenome, phylogenetic analysis

## Abstract

In this study, we sequenced and characterized the complete mitochondrial genome (mitogenome) of *Quasilineus sinicus* Gibson, 1990 (Heteronemertea, Nemertea) using Illumina sequencing technology. The circular mitogenome was 16,358 bp in length and comprised 22 transfer RNA genes, 13 protein-coding genes, and two ribosomal RNA genes. Its overall base composition included 20.82% A, 41.06% T, 26.68% G, and 11.44% C; in fact, the mitogenome had a high A + T content of 61.88%. Furthermore, our phylogenetic analysis demonstrated that Paleonemertea, Pilidiophora, and Hoplonemertea were monophyletic groups, and *Q. sinicus* was most closely related to *Iwatanemertes piperata*.

The phylum Nemertea consists of approximately 1280 identified species of invertebrate animals known as nemerteans. Most of these species are free-living animals in marine environments with bodies that are only a few millimeters wide (Kajihara et al. 2008). While the phylogenetic classification of Nemertea has been unclear for a long time, it has recently been classified into three classes: Palaeonemertea, Pilidiophora, and Hoplonemertea (Strand et al. 2019). *Quasilineus sinicus* Gibson, 1990 is a heteronemertean species (Pilidiophora: Heteronemertea) that resides in intertidal zones (Gibson 1990). It is characterized by three black and two orange longitudinal stripes on the dorsal side of its body, which is slender, cylindrical, or slightly flat and its body can be 190 mm in length and 2 mm in width (Sun SC 1995). In this study, we sequenced the complete mitochondrial genome (mitogenome) of *Q. sinicus* and investigated its taxonomical and phylogenetic relationships within the class Pilidiophora of the phylum Nemertea.

With regard to regulations of Natural Science Foundation of Hebei Province (reference number: C2020406016), permission was obtained to collect samples from Diaolou Bay following the research program of Hebei Provincial Department of Science and Technology. First, we collected the adult specimens of *Q. sinicus* from Diaolou Bay (Lingao, Hainan province, China; 19°55′20″N, 109°31′46″E). Their taxonomic statuses were confirmed by Prof. Shi-Chun Sun from Ocean University of China. Then the voucher specimens of *Q. sinicus* were deposited in the Institute of Evolution & Marine Biodiversity of Ocean University of China (Shi-Chun Sun, sunsc@ouc.edu.cn; voucher number, 20160414C1). Subsequently, the genomic DNA was extracted from a single specimen using the TIANamp Genomic DNA Kit (TIANGEN, Beijing, China; NO. DP304). Next, a DNA library was prepared using the NEB Next^®^ Ultra™ DNA Library Prep Kit (NEB, USA) and was sequenced on an Illumina NovaSeq 6000 platform. Consequently, approximately 15 Gb of paired-end reads (2 × 150 bp) were generated, and the mitogenome was assembled *de novo* using GetOrganelle (Jin et al. 2020) with approximately 300× average coverage. The annotation of transfer RNA (tRNA) genes was performed by tRNAscan-SE2.0 (http://lowelabucsc.edu/tRNAscan-SE/.) and ARWEN (http://130.235.244.92/ARWEN/). Positions of the protein-coding genes (PCGs) were determined using the online NCBI ORF Finder server (https://www.ncbi.nlm.nih.gov/orffinder/), additionally, these positions were manually validated by analyzing the BLAST (https://blast.ncbi.nlm.nih.gov/Blast.cgi) results of related species. The ribosomal RNA (rRNA) genes were annotated by aligning the rRNA gene sequences of species related to *Q. sinicus*. The genomic DNA sequence of *Q. sinicus* has been deposited in GenBank under the accession number MZ274345.

The complete circular mitogenome of *Q. sinicus* was 16,358 bp in length. Its overall nucleotide composition was 20.82% A, 41.06% T, 26.68% G, and 11.44% C. Similar to other nemertean species, nucleotide composition of the *Q. sinicus* mitogenome was strongly biased, as it had a total A + T content of 61.88%. In fact, the rRNA gene sequences had the highest A + T content (66.90%), followed by the tRNA gene sequences (65.33%) and PCG sequences (59.99%). Typically, the mitogenome contained 37 genes, including 22 tRNA genes, 13 PCGs, and two rRNA genes. Only two genes (*tRNA- Pro* and *tRNA- Thr*) were encoded on the light strain, whereas the other 35 genes were located on the heavy strain. Interestingly, all the PCG sequences had ATG as the start codon; the only exception was the *nad4* gene sequence that had GTG as the start codon. Stop codons included TAG (*cox1*, *cox2*, *cox3*, *nad2*, and *nad4L*), TAA (*nad3*, *nad4*, *atp6*, *atp8*, and *cytb*), and non-complete codons T- (*nad6*, *nad5*, and *nad1*) that are presumed to form a TAA codon upon post-transcriptional polyadenylation (Boore JL 2001). Twenty-one tRNA genes had a typical clover-leaf secondary structure, whereas *tRNA-Ser* (AGA) lacked a DHU arm; this loss was assumed to be an evolutionary loss (Haen et al. 2007). A 744 bp major non-coding region (mNCR) was located between *nad3* and *tRNA-Ser* (AGA) sequences. In addition, there is a 5 bp motif (AAAAG) which is repeated for 5 times in mNCR and this tandemly repeated sequences might play a central role in regulating the transcription process within genomes (Kolpakov et al. 2003). Furthermore, we identified other 29 relatively short intergenic regions (ranging from 1 to 203 bp) that were scattered across the mitogenome.

In order to investigate the phylogenetic relationships between *Q. sinicus* and other species in Nemertea, 20 mitogenomes of nemertean were firstly used for phylogenetic analyses, and *Katharina tunicate* and *Phoronopsis harmeri* were set as outgroups (Boore and Brown 1994; Chen et al. 2009; Podsiadlowski et al. 2009; Chen et al. 2011, 2012; Xu et al. 2012; Sun WY et al. 2014; Sun WY and Sun 2014; Shen et al. 2015; Shen and Sun 2016; Sun WY et al. 2016; Jiang and Deng 2018; Redak and Halanych 2019; Nam and Rhee 2020). Subsequently, a nucleotide concatenated dataset were generated using 13 PCGs and the best-fit model of nucleotide substitution of this concatenated dataset was estimated to be HKY + G using MrModeltest 2.2 (Nylander, 2004). Eventually, the phylogenetic analysis of Nemertea was reconstructed by MrBayes 3.2.2 (Miller et al. 2010). Our results revealed that Palaeonemertea, Pilidiophora, and Hoplonemertea were monophyletic groups (Figure 1). Notably, we discovered that *Q. sinicus* was closely related to *Iwatanemertes piperata*. As a kind of macrobenthos, *Q. sinicus* usually crawls on the seafloor sediment, which can increase the exchange of chemical substances at the sediment-water interface (Kanneworff and Christensen 1986). The present data will be useful for further phylogenetic studies and population genetic studies of this species.

**Figure 1.**
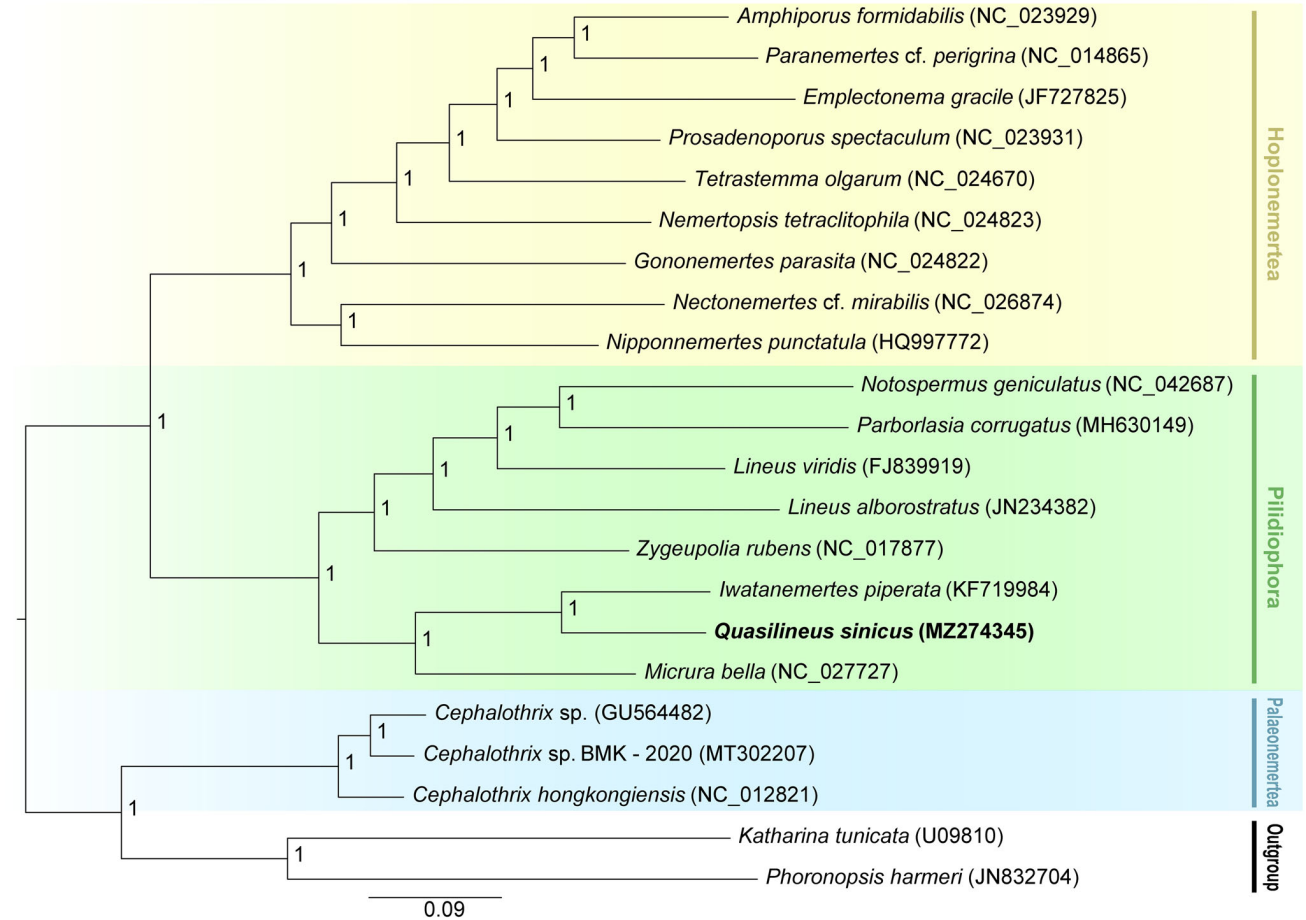
Phylogenetic tree shows evolutionary relationship among phylum Nemertea based on Bayesian Inference (BI) approach. Numbers behind major nodes denote posterior probabilities. The GenBank accession numbers are indicated on the right side of species names. The newly sequenced *Q*. *sinicus* mitogenome is highlighted using bold.

## Data Availability

The data that support the findings of this study are openly available in GenBank of NCBI at https://www.ncbi.nlm.nih.gov/, reference number MZ274345. The associated BioProject, Bio-Sample and SRA numbers are PRJNA799904, SAMN25211098 and SRS11750639, respectively.
